# Network Analytical Tool for Monitoring Global Food Safety Highlights China

**DOI:** 10.1371/journal.pone.0006680

**Published:** 2009-08-18

**Authors:** Tamás Nepusz, Andrea Petróczi, Declan P. Naughton

**Affiliations:** School of Life Sciences, Kingston University, London, United Kingdom; CIET, Canada

## Abstract

**Background:**

The Beijing Declaration on food safety and security was signed by over fifty countries with the aim of developing comprehensive programs for monitoring food safety and security on behalf of their citizens. Currently, comprehensive systems for food safety and security are absent in many countries, and the systems that are in place have been developed on different principles allowing poor opportunities for integration.

**Methodology/Principal Findings:**

We have developed a user-friendly analytical tool based on network approaches for instant customized analysis of food alert patterns in the European dataset from the Rapid Alert System for Food and Feed. Data taken from alert logs between January 2003 – August 2008 were processed using network analysis to i) capture complexity, ii) analyze trends, and iii) predict possible effects of interventions by identifying patterns of reporting activities between countries. The detector and transgressor relationships are readily identifiable between countries which are ranked using i) Google's PageRank algorithm and ii) the HITS algorithm of Kleinberg. The program identifies Iran, China and Turkey as the transgressors with the largest number of alerts. However, when characterized by impact, counting the transgressor index and the number of countries involved, China predominates as a transgressor country.

**Conclusions/Significance:**

This study reports the first development of a network analysis approach to inform countries on their transgressor and detector profiles as a user-friendly aid for the adoption of the Beijing Declaration. The ability to instantly access the country-specific components of the several thousand annual reports will enable each country to identify the major transgressors and detectors within its trading network. Moreover, the tool can be used to monitor trading countries for improved detector/transgressor ratios.

## Introduction

Food safety and security is a worldwide priority issue. In accordance with the Beijing Declaration, all signatory countries have agreed to develop comprehensive programs for monitoring food safety and security on behalf of their citizens. Market globalization, coupled with the information revolution, brings a number of challenges to monitoring food safety, such as comprehension and presentation of large and continuously growing (living) data sets. At the operational level, investigations are frequently necessary on data sets which are under daily or hourly expansion through a number of levels of complexity. Although some 5% of EU foodstuffs are recalled owing to contamination at source, the majority of alerts happen after export from checks at border crossings or during marketing [1]. While attempts have been made to regulate food safety at continental or global levels, rules and regulations are in effect at the local level (i.e. border control or market testing). Actions of individual counties, whether they are exporting or importing, are motivated by their own local interest, and they form the intricate pattern of the global food safety. This pattern is organically emerging from the individual actions and can only be studied with posteriori analyses using food alert logs.

Whilst food alert counts provide useful information, they tend to focus on a single element of this complex picture. Owing to the enormity and frequency of arrival of the data involved, the development of new monitoring systems is warranted to facilitate wider participation in food alerting and to provide early detection of potential ‘epidemics’ of contaminated foodstuffs (e.g. melamine in Chinese food products). The latter goal is particularly important when the reason for alert is a contamination that could endanger health, such as melamine, mycotoxins, nitrates, or heavy metals. There is a need to develop indicators that simultaneously take various factors into consideration such as the relationship between transgressors and detectors (i.e. in addition to the number of reports received, the model also takes the detector for each report into consideration), reason for food alerts, type of food (final product vs. ingredients) and time. Preliminary results from a small data set capturing 11 months in 2007 indicate that less than one dozen countries are major detectors in the food alert process [2], which currently suffers from a paucity of harmonisation and extensive inputs from limited participants. Countries with rigorous testing and reporting programs invest considerably in global food safety whereas other countries appear to be less equipped or concerned. The aim of this study was to extend our previous report on network analysis relating to food alerts [2] and develop a user-friendly analytical tool for ready access to food alert patterns that may in the future also be used as a searchable detection system for persistent producers of unsuitable foodstuffs. Targeted testing, informed by the proposed approach, will afford an increased likelihood of detecting foodstuff unsuitable for marketing and consumption.

## Results

**Figure 1:**
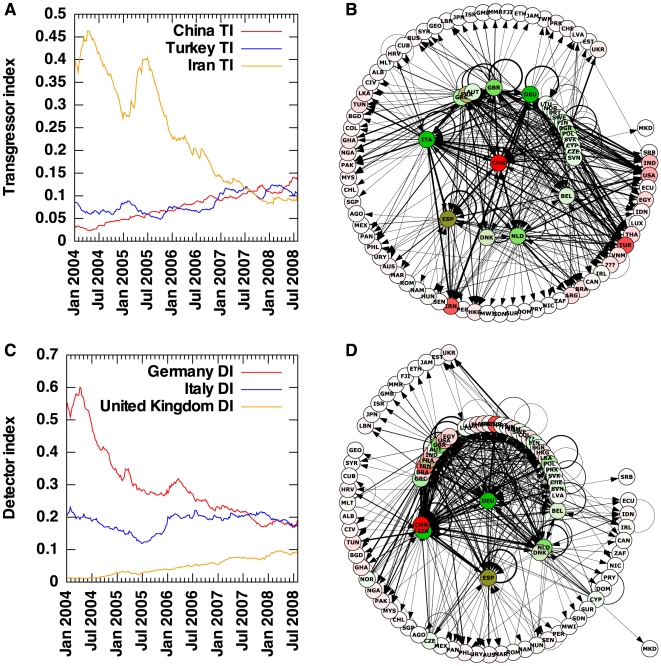
Transgressor and detector indices over time and network representations for selected countries. (A) Transgressor indices of China, Turkey and Iran from Jan 2004 to Jul 2008, derived from exponentially decaying edge weights. Plotted values are averaged over a 7-day window to improve readability. (B) The state of the food alert network on 1 July 2008, focusing on China (CHN). Edges with weight less than 1 are not shown. Arrows point from reporting countries to countries being reported on. Countries reporting on China or countries China reported on (currently none) are placed on the inner circle; countries not reporting directly on China but being connected to direct reporters on China are placed on the outer circle. Shades of red denote countries with high transgressor index; shades of green denote countries with high detector index. Edge thickness scales with the logarithm of the corresponding edge weight. (C) Detector indices of Germany, Italy and the United Kingdom from Jan 2004 to Jul 2008, derived from exponentially decaying edge weights. Plotted values are averaged as above. (D) The state of the food alert network on 1 July 2008, focusing on Germany (DEU). Arrowheads, edge thicknesses, vertex colors and layout as above.

We used two different algorithms to calculate the transgressor indices [TI] and detector indices [DI]: i) Google's PageRank algorithm [3] and ii) the HITS algorithm of Kleinberg [4]. Both measures are normalized so the sum of all TI and all DI separately is always equal to 1 at any given time as described under Methods. Our analytical tool is on open access via the Internet [http://staffnet.kingston.ac.uk/~ku36087/foodalert/]. Its interactive visualization application enables users to rapidly access information about the patterns of reports over a wide range of parameters using user selected durations. These include: reporting countries, reported countries, extent of reporting activity and networks in reporting at the country level. Our results are visualized in an interactive graph that makes all food report connections transparent at once while allowing the user to focus on a selected country at any given time. As shown in **Figure 1A**, the levels of reports against a country can be instantly plotted from the first alert against the given country to the selected end period.

**Figure 2:**
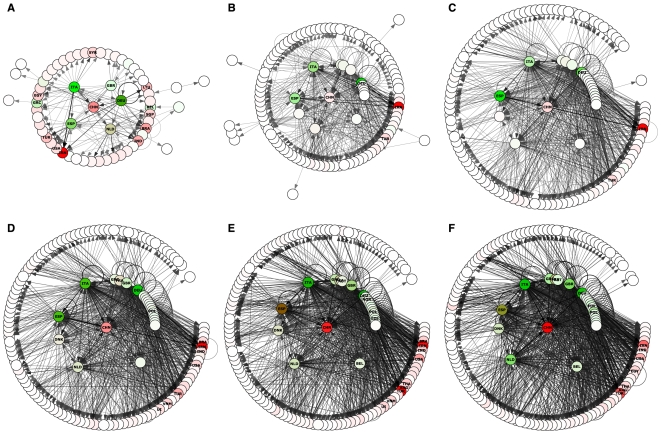
Snapshots of growth and changes in the network structures of the RASFF between 2003–2008, taken on the 1^st^ of July each year, focusing on China (in the center of the layout). (A). 2003. (B) 2004. (C) 2005. (D) 2006. (E) 2007. (F) 2008. Countries appearing after 1 Jan 2004 are as follows: Algeria (DZA), Angola (AGO), Honduras (HND), Jordan (JOR), Cambodia (KHM), Slovenia (SVN), Burkina Faso (BFA), Croatia (HRV), Jamaica (JAM), Guinea (GIN), Nepal (NPL), Gabon (GAB), Kazakhstan (KAZ), Malawi (MWI), Comoros (COM), Georgia (GEO), Afghanistan (AFG), Grenada (GRD), Greenland (GRL), Mozambique (MOZ), Haiti (HTI), Latvia (LVA), Fiji (FJI), Malta (MLT), San Marino (SMR), Costa Rica (CRI), Congo (COG), Iceland (ISL), Ethiopia (ETH), Niger (NER), Moldova (MDA), Guernsey (GGY), Maldives (MDV), Zambia (ZMB), Guatemala (GTM), Zimbabwe (ZWE), Oman (OMN). Macedonia (MKD), Ukraine (UKR), Swaziland (SWZ), Tonga (TON), Guyana (GUY), Bosnia and Herzegovina (BIH), Azerbaijan (AZE), Uzbekistan (UZB), Eritrea (ERI), Kuwait (KWT), Togo (TGO), Aruba (ABW), Sierra Leone (SLE), Monaco (MCO), Armenia (ARM), United Arab Emirates (ARE), Sudan (SDN), Papua New Guinea (PNG), Cuba (CUB).

**Figure 3:**
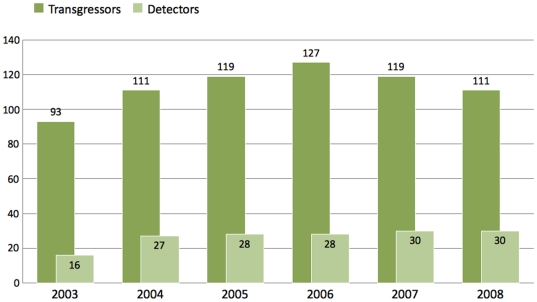
Change in the number of transgressor and detector countries over the six-year period. 2008 is pictured on alerts recorded until 23/08/2008. Note: there is an overlap between the two categories.

The growth and changes in global food alerts, as reported from an EU perspective, are illustrated in **Figure 2**. Food alert reports adopt an ‘infringement’ approach, focusing on the frequency and trends in reasons for food alert. Network analysis highlighted differences in the underlying structures of food alerts that otherwise would have remained hidden. As seen in **Figure 2B** [and **Table 1**], the number of alerts for each country frequently do not correspond to the impact on other countries as shown by the TI indices. For example, comparing China to Iran, the latter has the highest number of alerts but has a lower impact relative to the total transgressions over a given period. On the other hand, China has a major increase in alerts against its produce over the period as shown by annual sampling in **Figure 3**. The impact of transgressor countries is further highlighted when limiting the weight of edges taken into consideration from below [**Table 2**]. Although several transgressor countries have impact on some 25 detector countries with no cutoff, only China impacts on above ten detector countries when only edges with weight >5 are taken into account.

**Table 1:** The cumulative number of food alerts and transgressor indices (TI) indices for the countries listed among the first 30 in their category.

Country	Alerts	Country	TI (PageRank)	Country	TI (HITS)
IRN	1764.0	CHN	0.13998	CHN	0.08672
CHN	1305.0	TUR	0.10012	TUR	0.05693
TUR	1164.0	IRN	0.09248	USA	0.04348
USA	657.0	ESP	0.05934	ESP	0.03571
ESP	610.0	USA	0.04776	IRN	0.03051
DEU	577.0	IND	0.04033	FRA	0.02588
IND	568.0	FRA	0.03894	NLD	0.02564
FRA	480.0	DEU	0.03426	IND	0.02484
ITA	475.0	ITA	0.02989	DEU	0.02076
BRA	462.0	EGY	0.02243	POL	0.02028
THA	398.0	THA	0.02106	ARG	0.01740
VNM	329.0	ARG	0.02034	ITA	0.01739
UI	309.0	TUN	0.01802	UKR	0.01543
ARG	294.0	GBR	0.01744	THA	0.01487
GBR	289.0	UI	0.01674	VNM	0.01320
NLD	265.0	VNM	0.01564	BRA	0.01255
POL	256.0	NLD	0.01557	UI	0.01199
GHA	224.0	BRA	0.01550	GBR	0.01176
IDN	217.0	POL	0.01392	BEL	0.01053
DNK	208.0	DNK	0.01309	EGY	0.01011
EGY	178.0	HKG	0.01214	AUS	0.00969
BEL	149.0	GHA	0.01188	CAN	0.00948
GRC	137.0	LKA	0.01182	IDN	0.00905
NGA	134.0	NGA	0.01032	PAK	0.00876
HKG	133.0	GRC	0.00852	DNK	0.00762
PAK	115.0	BEL	0.00824	TUN	0.00750
UKR	103.0	PAK	0.00784	RUS	0.00743
PHL	102.0	UKR	0.00780	HKG	0.00726
BGD	101.0	PHL	0.00701	HUN	0.00709
MYS	94.0	IDN	0.00675	GRC	0.00709

UI denotes unidentified origin. “Alerts” means the total number of food alerts issued against that country up to 1 July 2008, “TI (PageRank)” and “TI (HITS)” are the two variants of the transgressor index based on the exponentially decaying edge weight model on 1 July 2008.

**Table 2:** Impact on countries by selected transgressors at edge-weight cut-off value of 0 and 5.

Transgressor Country	Detector countries (cutoff = 0)	Detector countries (cutoff = 5)
CHN	AUT, BEL, BGR, CYP, CZE, DEU, DNK, ESP, EST, FIN, FRA, GBR, GRC, HUN, ITA, LTU, LVA, MLT, NLD, NOR, POL, PRT, SVK, SVN, SWE	BEL, CZE, DEU, ESP, FIN, FRA, GBR, GRC, ITA, NLD, POL, SVN
IND	AUT, BEL, BGR, CYP, CZE, DEU, DNK, ESP, EST, FIN, FRA, GBR, GRC, IRL, ITA, LTU, LVA, MLT, NLD, NOR, POL, PRT, ROM, SVN, SWE, UI	BEL, DEU, GBR, ITA, POL
IRN	AUT, BEL, CYP, CZE, DEU, DNK, ESP, EST, FIN, FRA, GBR, GRC, HUN, ITA, LTU, LUX, LVA, MLT, NLD, NOR, POL, PRT, SVK, SVN, SWE	DEU, ESP, GRC, ITA
TUR	AUT, BEL, BGR, CYP, CZE, DEU, DNK, ESP, EST, FIN, FRA, GBR, GRC, HUN, IRL, ITA, LTU, LVA, MLT, NLD, NOR, POL, PRT, ROM, SVK, SVN, SWE, AUT, DEU, FRA, GBR, GRC, ITA, NLD, POL, SVK	AUT, DEU, FRA, GBR, GRC, ITA, NLD, POL, SVK
THA	BEL, CYP, CZE, DEU, DNK, ESP, EST, FIN, FRA, GBR, GRC, ISL, ITA, LVA, MLT, NLD, NOR, POL, PRT, SVK, SVN, SWE	BEL, DEU, FIN, GBR, NLD, NOR
USA	AUT, BEL, CYP, CZE, DEU, DNK, ESP, EST, FIN, FRA, GBR, GRC, HUN, IRL, ISL, ITA, LTU, LUX, LVA, MLT, NLD, NOR, POL, PRT, ROM, SVN, SWE, UI	AUT, BEL, DEU, ESP, FIN, GBR, GRC, ITA, NLD, SWE

Edge-weights are derived according to the exponentially decaying model. Countries are listed in alphabetical order, UI = unidentified origin (the Commission Services).

**Figure 4:**
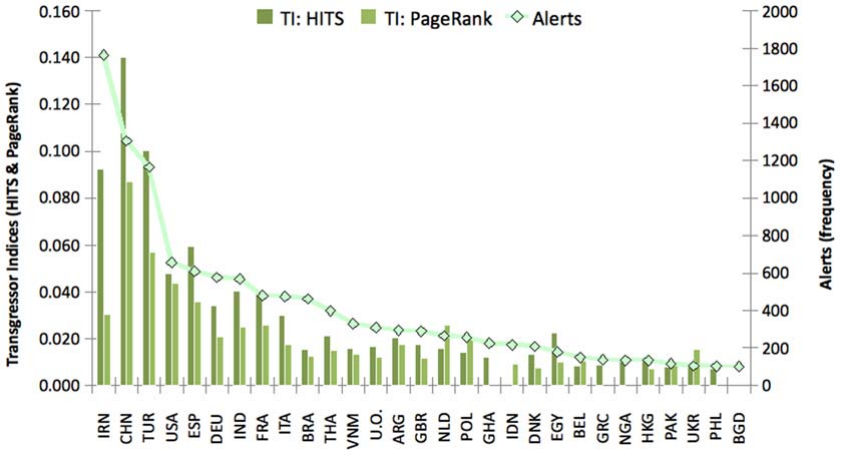
Comparison of the accumulated number of food alerts and the transgressor indices. The Y axis corresponds to the accumulated number of food alerts made against a product from a given country and the transgressor indices (TI) for the top 25 transgressors. The line shows the number of food alerts made between 1 January 2003 and 1 July 2008 against the given countries. Bars represent the transgressor indices using PageRank and HITS, calculated with exponential weighting for 1 July 2008. Corresponding data are shown in Table 1.

Although countries' ranks on the three lists [**Table 1** and **Figure 4**] showed significant correlation (Kendall tau = 0.76, p = 1.19×10^−7^; 0.66, p = 3.69×10^−6^; and 0.64, p = 8.94×10^−6^ for pairs of *Alerts – HITS*, *Alerts – PageRank* and *HITS - PageRank*, respectively for the top 30 countries), there was a notable difference between volume (number of alerts) and impact (quantified by the TI indices and the number of countries involved) for some food producer countries. Whilst the number of food alerts appears to level off after 2006 with no significant seasonal variation, the number of countries involved in the food alert system has grown from 94 to 151. Although, based on the TI/DI values, the new countries appear to be insignificant transgressors, their appearance has contributed to the complexity of global food alerts. Thus, whilst the numbers of alerts are relatively easy to compare, obtaining information on impact requires a network approach. The importance of having information on the latter aspect is underscored by highly concerning incidents, such as the recently discovered melamine contamination in Chinese milk and milk-based products; or the *Salmonella* contamination of peanut butter and related products in multiple countries in 2009. The latter incidents have resulted in a critical evaluation of the currently disjoint US food safety system. In keeping with the increasing complexity, the intention is to modernise the system by adding the ability to handle complex information from multiple sources and implement preventive measures [5].

## Discussion

The approach taken for this project from the EU perspective could complement the work of the International Food Safety Authorities Network (INFOSAN) to assist researchers and provide information on food safety in relation to exporting countries for interested individuals. Our approach highlights the advantage of the network approach over simple frequency counts in that it takes into consideration not only the number of reports received by the transgressor country but also the number of reporting countries (detectors) related to alerts from the transgressor. The results can be obtained and downloaded as figures for any country for any selected time period starting from the first alert. Our unbiased program provides stakeholders (policy makers, health and food safety authorities and researchers) with a systematic, rigorous but user-friendly approach to i) capture complexity, ii) analyze trends, and iii) predict possible effects of interventions.

The usefulness of moving from simple frequency counts to a more complex methodology cannot be illustrated with a better example than the groundbreaking work on extending the simple “citation and backlinks to a webpage” method into a more informative measure, namely Google's PageRank [3]. Using a similar approach to characterize a country's behavior regarding food safety, we developed two indices (the transgressor and the detector indices, denoted by TI and DI, respectively) that quantify the extent and role of involvement of a country in global food safety. High TI means that numerous alerts are issued against that country by others, while high DI denotes countries that issued many food alerts against food products from other countries.

This study reports the first development of a network visualization approach to inform countries on their transgressor and detector profiles as a user-friendly aid for the adoption of the Beijing Declaration. The ability to instantly access the country-specific components of the several thousand annual reports will enable each country to identify the major transgressors and detectors within its trading network. Moreover, the tool can be used to monitor trading countries for improved detector/transgressor ratios. Our program allows facile handling of enormous quantities of data that arise from food alerts and recalls in line with the needs of countries that are adopting and implementing food security measures. Future developments will include an optional filter by reasons for alerts and in case of contaminations, an optional display of information on the amount of contamination found in foodstuff that triggered the alert. These extensions may be used for monitoring purposes. The data behind the visualization tool is a living data set, currently spans from 2003 to 2008, but is expandable as new reports arrive. The next major step in our approach is to develop and implement a data interchange format between food alert systems and agencies which should include standardization. Visualization in real time arising from a standardized data interchange format would enable public health agencies and researchers to process food alerts as they are issued from multiple agencies.

## Methods

The data used for the analyses presented in this report were taken from Rapid Alert System for Food and Feed (RASFF) logs between January 2003 and August 2008. Thus the results are from an EU perspective and do not amalgamate data from parallel food alert systems such as in the USA and Australia. Thus, these countries appear only as transgressors in the RASFF system. The study did not require ethical approval.

### Network representation of food alerts

Our earlier work focused on summarizing food alerts in a simple and concise network representation. In the present context, a food alert is considered to be a formal warning issued by a *reporting* country on another country regarding some faulty foodstuff that is believed to originate from that country. Every alert has a unique issue date assigned to it and we make use of the identity of the two countries involved and the issue date when we derive the network of food alerts.

The network representation is composed of vertices (representing countries) and edges (representing food alerts). Edges are directed and always point from a *reporting* country to a country being *reported on*. Edges also have *weights*, capturing the intensity of food alerts between the two countries at the two endpoints of the edge. In the simplest case, the weight of an edge pointing from country A to country B is the number of alerts A issued on B within the considered time frame. Based on this network representation, we can derive numeric scores for each country to describe their roles within the network; e.g., the total weight of outgoing edges adjacent to a given node may denote how actively that country participates in the detection of hazardous foodstuff. The exact scores we used will be described later.

Food alerts are issued almost every day, thus it is reasonable to assume that the network is not static; it evolves in time as new alerts are issued. If we want to obtain an accurate picture of the network at a given time instance *t*, we must consider all alerts issued up to *t* and derive edge weights in a way that considers not only the *number* of food alerts between two given countries but also the *time* when those alerts were issued. Intuitively, more recent food alerts should be taken into account with a larger weight than those that have occurred months ago. From now on, we assume that time is measured in days, thus *t* denotes the number of days that have passed since the day of the first food alert in our dataset. The weight of a single food alert issued at time instance *t_0_* will be assigned a weight of *λ* ^t−t0^ at time instance *t* if *t*> = *t_0_* and zero if *t*<*t_0_*. *λ* is an arbitrary positive constant that is strictly less than 1. This means that the weight of a food alert is exactly 1 on the day it is issued, *λ* one day later, *λ* ^2^ two days later, *λ^k^ k* days later and so on. In other words, the weight of a food alert decays exponentially as time passes and the rate of decay is controlled by *λ*. The weight of an edge from country A to country B at time instance *t* is then simply defined as the sum of the weights of all food alerts issued by country A on country B at time instance *t*. This way, we take into account both the number and the age of food alerts when deriving edge weights. In the calculations described in this paper, we used *λ = 0.5^1/180^ = 0.9961* as this means that the weight of a food alert is halved every 180 days.

### Transgressor and detector indices

For each country in the network, we will define two indices: the transgressor and the detector index (TI and DI, respectively). The transgressor index of a country is high if many alerts are issued against that country by other countries, while the detector index is high if the country issues many useful food alerts against other countries. Since there is no baseline value against which we can assess individual countries, we normalize the indices to ensure that the sum of the both the transgressor indices and the detector indices over all the countries equals 1. We consider two different approaches, inspired by two well-established data mining techniques: the HITS algorithm of Kleinberg [4] and the PageRank algorithm of Brin & Page [3].

### Transgressor and detector indices based on the HITS algorithm

The basic idea of this algorithm can be formulated in the domain of food alerts as follows: a country should have a high transgressor index if there are many reports on this country issued by countries having a high detector index; similarly, a country should have a high detector index if this country issues many reports against countries with high transgressor indices. Formally, let *α_i_* denote the transgressor and *β_i_* denote the detector index of country *i*. Let *w_ij_* be the weight of the edge from country *i* to country *j* (*w_ij_* is zero if there is no edge from country *i* to country *j*). The indices can be calculated by the following procedure:

1. Start from arbitrary *α_i_* and *β_i_* values.
2. Let 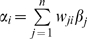

3. Let 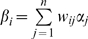

4. Normalize *α_i_* to make its sum exactly 1.
5. Normalize *β_i_* to make its sum exactly 1.
6. Go back to step 2 until the process converges.

### Transgressor and detector indices based on the PageRank algorithm

The PageRank algorithm calculates transgressor and detector indices separately. The key idea here is based on a random walk over the vertices and edges of the network. A random walker starts from any arbitrary vertex, and in each step, it either chooses one of the outgoing edges of that vertex and jumps to the other endpoint of that edge with probability *γ*, or jumps to another randomly selected vertex with probability 1-*γ*. *γ* is typically set to 0.85 and the probability of choosing an outgoing edge is proportional to the weight of that edge. Vertices that have many incoming and few outgoing edges are harder to escape from, therefore the probability of being at vertex *i* after infinitely many steps is a suitable transgressor index for that vertex. 1- *γ* denotes the probability of taking a completely random jump on the network, and it is necessary for the random walker to escape from nodes having no outgoing edges. With γ = 1, the random walker could be stuck forever in such “sink” vertices. The exact formula for calculating *PR_i_* (the PageRank score of vertex *i*) is as follows:
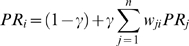



The equation system above can also be solved iteratively and it always converges to a unique solution that is independent from the initial conditions. Similar reasoning shows that the PageRank scores of the network that is obtained from the original one by reversing all the edges is a suitable detector index.
